# Resource use by patients hospitalized with community-acquired pneumonia in Europe: analysis of the REACH study

**DOI:** 10.1186/1471-2466-14-36

**Published:** 2014-03-05

**Authors:** Helmut Ostermann, Javier Garau, Jesús Medina, Esther Pascual, Kyle McBride, Francesco Blasi

**Affiliations:** 1 Department of Internal Medicine III, Haematology and Oncology, University Hospital Munich, Munich, Germany; 2 Department of Medicine, Hospital Universitari Mutua de Terrassa, Plaza Doctor Robert 5, 08221 Terrassa, Barcelona, Spain; 3 Medical Evidence Centre, Global Medical Affairs, AstraZeneca, Parque Norte, Edificio Roble, Serrano Galvache 56, 28033 Madrid, Spain; 4 Medical Department, Clinical Research Unit, AstraZeneca, Parque Norte, Edificio Roble, Serrano Galvache 56, 28033 Madrid, Spain; 5 Instat Services, Inc., 1 Wilson Street, Chatham, NJ 07928, USA; 6 Department of Pathophysiology and Transplantation, Università degli Studi di Milano, IRCCS Fondazione Ca’ Granda, Ospedale Maggiore Policlinico, Milan, Italy

**Keywords:** Anti-bacterial agents, Community-acquired pneumonia, Economics, Medical, Retrospective studies

## Abstract

**Background:**

Management of community-acquired pneumonia (CAP) places a considerable burden on hospital resources. REACH was a retrospective, observational study (NCT01293435) involving adults ≥18 years old hospitalized with CAP and requiring in-hospital treatment with intravenous antibiotics conducted to collect data on current clinical management patterns and resource use for CAP in hospitals in ten European countries.

**Methods:**

Data were collected via electronic Case Report Forms detailing patient and disease characteristics, microbiological diagnosis, treatments before and during hospitalization, clinical outcomes and health resource consumption.

**Results:**

Patients with initial antibiotic treatment modification (n = 589; 28.9%) had a longer mean hospital stay than those without (16.1 [SD: 13.1; median 12.0] versus 11.1 [SD: 8.9; median: 9.0] days) and higher ICU admission rate (18.0% versus 11.9%). Septic shock (6.8% versus 3.0%), mechanical ventilation (22.2% versus 9.7%), blood pressure support (fluid resuscitation: 19.4% versus 11.4%), parenteral nutrition (6.5% versus 3.9%) and renal replacement therapy (4.2% versus 1.4%) were all more common in patients with treatment modification than in those without. Hospital stay was longer in patients with comorbidities than in those without (mean 13.3 [SD: 11.1; median: 10.0] versus 10.0 [SD: 7.5; median: 8.0] days).

**Conclusions:**

Initial antibiotic treatment modification in patients with CAP is common and is associated with considerable additional resource use. Reassessment of optimal management paradigms for patients hospitalized with CAP may be warranted.

## Background

In Europe, the total annual costs of pneumonia exceed €10 billion [1]. Community-acquired pneumonia (CAP), with an annual incidence rate of between 1.6 and 10.8 cases per 1,000 adults per year [2], makes a considerable contribution to this figure. In Spain, there are reported to be 51,000 hospitalizations for CAP per year (a rate of 1.6 per 1,000 population) [3], while median total costs per patient for hospitalized CAP patients in Germany are estimated at US$1,333 (2003 costs) [2].

Studies in the USA show that the main component of the economic burden of CAP is inpatient treatment costs, which account for around 90% of the total cost [4]. Furthermore, of these costs, hospital stay and antibiotic treatment are the largest contributors [2,5]. These components are also interlinked, in that length of stay is influenced by choice of initial-line antibiotic; inappropriate therapy results in additional costs [6]. While these data are valuable, there are no comparable or more comprehensive data on the economic burden of CAP across Europe as a whole and the contribution of hospital resource use to this burden.

The REACH study (Retrospective Study to Assess the Clinical Management of Patients With Moderate-to-Severe complicated skin and skin structure infections [cSSTI] or CAP in the Hospital Setting) was conducted to address the gaps in the available data. The main objectives of the study, which are reported in the primary publication for this study [7], were to collect detailed background data on the population of patients hospitalized with CAP in Europe, to provide a summary of clinical practice decisions in these patients and to understand the impact of these decisions in terms of rates of initial antibiotic treatment modification and mortality. A key secondary objective was to gather data on resource use in patients hospitalized with CAP and the associated costs to understand the economic impact of the disease. These health economic data are reported here, including an assessment of inter-country differences across the region. The complicated skin and soft tissue infections data were considered and are reported separately.

## Methods

### Overview

REACH was a multinational, multicentre, observational, retrospective cohort study of patients hospitalized with CAP and cSSTI (NCT01293435; cSSTI data are analysed and reported separately). Patients were enrolled from 128 sites in ten participating countries (Belgium, France, Germany, Greece, Italy, the Netherlands, Portugal, Spain, Turkey and the UK). All included patients were hospitalized between March 2010 and February 2011. Study design and patient inclusion and exclusion criteria are summarised in the companion paper presenting the results of the primary objectives [7]. Study variables were collected via an electronic Case Report Form. In brief, the study collected data about patient demographics, disease characteristics and diagnosis, management (with particular focus on antibiotics received), clinical outcomes (including initial treatment modification rate) and use of resources. The study was performed according to Good Clinical Practice and the Declaration of Helsinki. All local ethics committees approved the study protocol (a list of all participating sites can be found in Additional file 1). Local legislation relating to written informed consent for non-interventional studies was followed in each country; in Germany and Portugal, where this information is mandatory, written informed consent was collected.

### Statistical methods and data interpretation

This was a retrospective non-interventional study, using a descriptive analysis approach to assess clinical management, clinical outcomes and healthcare resource use. All calculations and summaries were produced using SAS Version 9.2. Only descriptive (no analytical) data are provided.

Hospitalization costs for each of the countries involved were determined using estimated unit cost values for primary and secondary healthcare services derived from the World Health Organization (WHO) CHOosing Interventions that are Cost-Effective (CHOICE) project (http://www.who.int/choice/country/country_specific/en/index.html) [8]. The values are averages of unit costs for the country, based on specific assumptions regarding the organisation of health services and operational capacity.

‘Initial antibiotic treatment modification’ was defined as a change in initial antibiotic treatment due to insufficient response, adverse reaction, interaction with other drugs, non-suitability of the initial antibiotic based on the results of microbiological tests or changes to or addition of new agents in a subsequent line, alone or in combination. Comorbidities were defined as relevant medical conditions at hospitalization. Investigators could select from a list of comorbidities outlined in the companion manuscript [7], or include other conditions based on their own medical criteria. Recurrences were defined as patients who were hospitalized again (due to CAP), after initial discharge. Immunosuppressed/immunocompromised patients were patients who were on haemodialysis or chemotherapy, with neutropenia, stem cell transplantation, HIV/AIDS or iatrogenic immunosuppression (patients on biological therapy) or corticoids (15 mg/day for ≥ 14 days, or equivalent dose). The requirement for isolation was based on the investigator’s interpretation.

## Results

### Patient population

The analysis population included 2,039 patients. The majority of patients (78.8%; n = 1,607) had CAP only (as defined by residence in a private house or apartment prior to admission) while 12.0% (n = 245) of patients had healthcare-associated pneumonia (HCAP; defined as patients with residence in a nursing home, or receiving home care through a healthcare agency, or admitted to hospital in the 3 months prior to index admission, or undergoing haemodialysis, or receiving chemotherapy for active cancer). A detailed breakdown of information on the study population is provided in the companion paper [7].

### Clinical outcomes

Clinical outcomes data for the full analysis population are given in the primary publication [7]. Initial treatment modification occurred in 28.9% of patients (n = 589). The most common reasons for initial antibiotic treatment modification were insufficient response to treatment (12.0%) and adverse events (2%). The mean time to treatment modification in the total population was 5.0 days (standard deviation [SD]: 3.8; median: 4.0; n = 760). The mean time to clinical stability was 5.6 days (SD: 5.1; median: 4.0; n = 1,603). Death occurred in 7.2% of patients (n = 147). In 5.1% of patients, streamlining of therapy, defined as de-escalation to narrower spectrum antibiotic in response to patient improvement or confirmed microbiological diagnosis, was undertaken; this was not counted as initial antibiotic treatment modification.

Clinical outcomes data by country are shown in Table 1. Particularly high initial antibiotic treatment modification rates were observed in the UK (37.7%; n = 43/114) and in Belgium (35.6%; n = 68/191), while low initial antibiotic treatment modification rates were observed in France (15.6%; n = 57/366) and Greece (21.9%; n = 47/215).

**Table 1:** Clinical outcomes by country

**Country**	**Number of patients, n (%)**	**Initial antibiotic treatment modification, n (%)**	**Mortality, n (%)**	**Time to clinical stability, days, mean**
Belgium	191 (9.4)	68 (35.6)	23 (12.0)	6.0 (n = 135)
France	366 (17.9)	57 (15.6)	19 (5.2)	4.4 (n = 277)
Germany	50 (2.5)	18 (36.0)	0	5.9 (n = 35)
Greece	215 (10.5)	47 (21.9)	5 (2.3)	5.6 (n = 142)
Italy	300 (14.7)	100 (33.3)	3 (1.0)	6.3 (n = 263)
The Netherlands	203 (10.0)	69 (34.0)	22 (10.8)	4.6 (n = 174)
Portugal	121 (5.9)	35 (28.9)	19 (15.7)	6.0 (n = 65)
Spain	279 (13.7)	96 (34.4)	19 (6.8)	6.0 (n = 247)
Turkey	200 (9.8)	56 (28.0)	17 (8.5)	7.2 (n = 171)
United Kingdom	114 (5.6)	43 (37.7)	20 (17.5)	3.9 (n = 94)
**Total population**	**2,039 (100)**	**589 (28.9)**	**147 (7.2)**	**5.6 (n = 1,603)**

### Hospital stay and resource use

Hospital stay and resource use for the full analysis population (N = 2,039) and by disease characteristics at baseline are shown in Table 2. The mean length of stay in hospital was 12.6 days (SD: 10.6; median: 10.0), with 13.6% of patients admitted to the intensive care unit (ICU), where they stayed for a mean of 9.5 days (SD: 11.7; median: 5.0). The reason for admission was not given. Similar percentages of patients required fluid resuscitation and mechanical ventilation, and mechanical ventilation was invasive in approximately half of the ventilated patients (the remainder receiving non-invasive mechanical ventilation). Acute renal failure occurred in 2.3% of patients; it is unknown whether these occurrences were treatment-related.

**Table 2:** Hospital stay and resource use (full analysis population and by disease characteristics)

**Resource**	**Full population (n = 2,039)**	**Disease characteristics**		
		**CAP (n = 1,607)**	**HCAP (n = 245)**	**Immunosuppressed/Immunocompromised (n = 72)**
Total duration of hospitalization*, days, mean (SD) [median]	12.6 (10.6) [10.0] (n = 1,978)	12.4 (10.4) [9.0] (n = 1,558)	13.2 (10.6) [11.0] (n = 235)	16.0 (13.3) [12.0] (n = 71)
Admitted to ICU at any time, n (%)	278 (13.6)	218 (13.6)	31 (12.7)	15 (20.8)
Time in ICU, days, mean (SD) [median]	9.5 (11.7) [5.0] (n = 244)	9.9 (12.3) [6.0] (n = 195)	5.2 (5.0) [2.5] (n = 28)	10.3 (12.0) [4.5] (n = 12)
Blood pressure support during hospitalization, n (%)				
Fluid resuscitation	251 (12.3)	178 (11.1)	43 (17.6)	15 (20.8)
Vasopressors	101 (5.0)	79 (4.9)	11 (4.5)	6 (8.3)
Invasive procedures	30 (1.5)	28 (1.7)	0	0
Isolation required, n (%)	154 (7.6)	99 (6.2)	23 (9.4)	19 (26.4)
Mechanical ventilation required during hospitalization, n (%)	280 (13.7)	215 (13.4)	36 (14.7)	11 (15.3)
Invasive	139 (6.8)	110 (6.8)	13 (5.3)	8 (11.1)
Duration, days, mean (SD) [median]	10.5 (12.7) [6.0] (n = 133)	10.4 (12.9) [6.0] (n = 126)	N/A	N/A
Non-invasive	166 (8.1)	125 (7.8)	26 (10.6)	4 (5.6)
Duration, days, mean (SD) [median]	5.2 (4.5) [4.0] (n = 155)	5.0 (4.6) [4.0] (n = 144)	N/A	N/A
Parenteral nutrition, n (%)	94 (4.6)	60 (3.7)	26 (8.2)	6 (8.3)
Duration of parenteral nutrition, days, mean (SD) [median]	9.1 (10.6) [5.0] (n = 88)	9.5 (11.4) [5.0] (n = 55)	6.6 (6.8) [5.0] (n = 19)	4.3 (2.5) [4.0] (n = 6)
Acute renal failure necessitating renal replacement therapy, n (%)	46 (2.3)	38 (2.4)	5 (2.0)	1 (1.4)
Duration of renal failure, days, mean (SD) [median]	6.5 (8.6) [3.0] (n = 37)	6.2 (7.1) [4.0] (n = 31)	11.5 (18.3) [2.5] (n = 4)	1.0 (-) [1.0] (n = 1)
Septic shock during hospitalization^†^, n (%)	84 (4.1)	61 (3.8)	9 (3.7)	8 (11.1)
Home-based care, n (%)	73 (3.6)	46 (2.9)	22 (9.0)	3 (4.2)

*Includes duration of all hospitalizations for patients with recurrences.

^†^Septic shock was defined as the presence of severe sepsis and one of the following conditions: a) systemic mean blood pressure of <60 mmHg (<80 mmHg if previous hypertension) after 20 to 30 mL/kg starch or 40 to 60 mL/kg serum saline solution; b) pulmonary capillary wedge pressure between 12 and 20 mmHg and need for dopamine of >5 mcg/kg/min; c) norepinephrine or epinephrine to maintain mean blood pressure at >60 mmHg (80 mmHg if previous hypertension).

CAP: community-acquired pneumonia; HCAP: healthcare-associated pneumonia; ICU: intensive care unit; SD: standard deviation.

Resource use was generally greater in patients with HCAP (n = 245) than with CAP (n = 1,607), including rates of fluid resuscitation, requirement for isolation and parenteral nutrition, and duration of renal failure. However, the duration of ICU stay and duration of parenteral nutrition were longer in patients with CAP than in those with HCAP. Immunosuppressed/immunocompromised patients with CAP had higher resource use compared with CAP only and HCAP patients.

Analyses of hospital stay and resource use by clinical outcomes are shown in Table 3. Patients requiring initial antibiotic treatment modification (n = 589) had a longer duration of hospital stay and were more likely to be admitted to the ICU, with a longer mean stay in the ICU than those not requiring modification (n = 1,387). Blood pressure support, mechanical ventilation, parenteral nutrition and renal replacement therapy were all also more commonly required by these patients.

**Table 3:** Hospital stay and resource use analysed by clinical outcomes

**Resource**	**Initial antibiotic treatment modification**		**Comorbidities**		**Recurrences**		**Septic shock**	
	**With (n = 589)**	**Without (n = 1,450)**	**With (n = 1,598)**	**Without (n = 441)**	**With (n = 94)**	**Without (n = 1,945)**	**With (n = 84)**	**Without (n = 1,955)**
Total duration of hospitalization, days, mean (SD) [median]	16.1 (13.1) [12.0] (n = 581)	11.1 (8.9) [9.0] (n = 1,397)	13.3 (11.1) [10.0] (n = 1,555)	10.0 (7.5) [8.0] (n = 423)	25.1 (16.9) [19.0] (n = 94)	11.5 (8.7) [9.0] (n = 1,548)	21.8 (18.7) [17.0] (n = 83)	12.2 (9.9) [9.0] (n = 1,895)
Admitted to ICU at any time, n (%)	106 (18.0)	172 (11.9)	219 (13.7)	59 (13.4)	19 (20.2)	177 (11.4)	69 (82.1)	209 (10.7)
Time in ICU, days, mean (SD) [median]	11.2 (13.6) [5.0] (n = 90)	8.5 (10.4) [5.5] (n = 154)	9.9 (12.5) [6.0] (n = 191)	8.3 (8.1) [5.0] (n = 53)	12.9 (17.8) [4.0] (n = 17)	8.7 (10.7) [5.0] (n = 176)	13.7 (16.2) [8.5] (n = 54)	8.3 (9.8) [5.0] (n = 190)
Blood pressure support during hospitalization, n (%)								
Fluid resuscitation	95 (16.1)	156 (10.8)	209 (13.1)	42 (9.5)	15 (16.0)	155 (10.0)	63 (75.0)	188 (9.6)
Vasopressors	46 (7.8)	55 (3.8)	77 (4.8)	24 (5.4)	5 (5.3)	42 (2.7)	71 (84.5)	30 (1.5)
Invasive procedures	13 (2.2)	17 (1.2)	22 (1.4)	8 (1.8)	2 (2.1)	13 (0.8)	12 (14.3)	18 (0.9)
Isolation required, n (%)	55 (9.3)	99 (6.8)	114 (7.1)	40 (9.1)	5 (5.3)	92 (5.9)	20 (23.8)	134 (6.9)
Mechanical ventilation required during hospitalization, n (%)	114 (19.4)	166 (11.4)	232 (14.5)	48 (10.9)	10 (10.6)	149 (9.6)	68 (81.0)	212 (10.8)
Invasive	61 (10.4)	78 (5.4)	108 (6.8)	31 (7.0)	4 (4.3)	56 (3.6)	60 (71.4)	79 (4.0)
Duration, days, mean (SD) [median]	12.9 (15.8) [8.0] (n = 58)	8.6 (9.3) [5.0] (n = 75)	10.9 (13.7) [6.0] (n = 103)	9.0 (8.1) [8.0] (n = 30)	21.3 (28.0) [9.5] (n = 4)	11.1 (9.9) [8.0] (n = 53)	10.8 (10.5) [8.0] (n = 58)	10.2 (14.2) [5.0] (n = 75)
Non-invasive	68 (11.5)	98 (6.8)	146 (9.1)	20 (4.5)	8 (8.5)	102 (6.6)	20 (23.8)	146 (7.5)
Duration, days, mean (SD) [median]	6.0 (5.4) [4.0] (n = 64)	4.6 (3.7) [4.0] (n = 91)	5.3 (4.7) [4.0] (n = 139)	4.1 (3.4) [3.5] (n = 16)	3.0 (0.8) [3.0] (n = 8)	5.6 (4.8) [4.0] (n = 99)	4.9 (5.1) [3.0] (n = 20)	5.2 (4.5) [4.0] (n = 135)
Parenteral nutrition, n (%)	38 (6.5)	56 (3.9)	79 (4.9)	15 (3.4)	5 (5.3)	50 (3.2)	16 (19.0)	78 (4.0)
Duration of parenteral nutrition, days, mean (SD) [median]	13.4 (14.9) [6.0] (n = 34)	6.4 (5.2) [5.0] (n = 54)	9.2 (10.7) [5.0] (n = 73)	8.4 (10.7) [4.0] (n = 15)	16.4 (22.9) [8.0] (n = 5)	8.0 (7.0) [5.0] (n = 46)	11.5 (13.9) [5.0] (n = 14)	8.6 (9.9) [5.0] (n = 74)
Acute renal failure necessitating renal replacement therapy, n (%)	25 (4.2)	21 (1.4)	36 (2.3)	10 (2.3)	1 (1.1)	16 (1.0)	22 (26.2)	24 (1.2)
Duration of renal failure, days, mean (SD) [median]	8.5 (8.6) [6.0] (n = 19)	4.3 (8.3) [2.0] (n = 18)	5.9 (7.4) [3.0] (n = 29)	8.6 (12.3) [2.5] (n = 8)	3.0 (-) [3.0] (n = 1)	6.8 (9.4) [3.5] (n = 14)	7.1 (8.9) [4.0] (n = 17)	6.0 (8.5) [3.0] (n = 20)
Septic shock, n (%)	40 (6.8)	44 (3.0)	61 (3.8)	23 (5.2)	4 (4.3)	34 (2.2)	N/A	N/A
Home-based care, n (%)	25 (4.2)	48 (3.3)	64 (4.0)	9 (2.0)	7 (7.4)	65 (4.2)	3 (3.6)	70 (3.6)

ICU: intensive care unit; SD: standard deviation.

Patients with comorbidities (n = 1,598), which included respiratory disease, diabetes and congestive heart disease, experienced longer stays in both hospital and ICU than those without (n = 441). Patients with recurrent CAP (n = 94) required more resources than patients with a single infectious episode (n = 1,945), with a higher length of hospital stay, rate of admission to ICU and longer stay once admitted. The duration of parenteral nutrition was more than doubled in patients with recurrent infection compared with those without.

As expected, patients with septic shock (n = 84) consumed more resources compared with those without (n = 1,955), with particularly high rates of blood pressure support, mechanical ventilation, parenteral nutrition and renal replacement therapy required in patients with septic shock than in those without.

A comparison of resource use patterns by participating country is shown in Table 4. The mean duration of hospital stay varied between 9.6 days (SD: 6.4; median: 7.0) in Greece and 15.0 days (SD: 13.2; median 11.0) in Belgium. Wide variation between countries was observed in the percentage of patients admitted to the ICU, with Belgium having the highest rate (35.6%; n = 68/191) and Italy the lowest (3.3%; n = 10/300). The mean duration of ICU stay was similar in the majority of countries, with the exception of Germany (22.5 days), although this finding was based on a very small sample size (n = 6/50). Blood pressure support in the form of fluid resuscitation was considerably more common in the UK than in other countries, while use of fluid resuscitation was highest in the Netherlands and Greece.

**Table 4:** Hospital stay and resource use analysed by country

**Resource**	**Full population (n = 2,039)**	**Country**									
		**Belgium (n = 191)**	**France (n = 366)**	**Germany (n = 50)**	**Greece (n = 215)**	**Italy (n = 300)**	**The Netherlands (n = 203)**	**Portugal (n = 121)**	**Spain (n = 279)**	**Turkey (n = 200)**	**UK (n = 114)**
Total duration of hospitalization, days, mean (SD) [median]	12.6 (10.6) [10.0] (n = 1,978)	15.0 (13.2) [11.0] (n = 190)	13.6 (11.5) [11.0] (n = 319)	11.4 (10.6) [9.0] (n = 48)	9.6 (6.4) [7.0] (n = 212)	13.1 (8.5) [11.0] (n = 298)	12.8 (14.0) [9.0] (n = 201)	13.6 (11.3) [10.0] (n = 121)	12.3 (9.6) [9.5] (n = 278)	12.4 (9.3) [10.0] (n = 197)	10.4 (9.4) [7.0] (n = 114)
Admitted to ICU at any time, n (%)	278 (13.6)	68 (35.6)	82 (22.4)	6 (12.0)	9 (4.2)	10 (3.3)	21 (10.3)	12 (9.9)	38 (13.6)	21 (10.5)	11 (9.6)
Time in ICU, days, mean (SD) [median]	9.5 (11.7) [5.0] (n = 244)	7.3 (8.7) [4.0] (n = 65)	9.9 (12.5) [7.0] (n = 75)	22.5 (31.5) [3.5] (n = 6)	16.4 (13.1) [9.0] (n = 9)	13.4 (12.1) [8.0] (n = 8)	8.8 (10.1) [4.0] (n = 15)	11.7 (9.3) [7.0] (n = 11)	8.4 (10.6) [4.5] (n = 36)	5.9 (4.2) [5.0] (n = 11)	11.1 (12.1) [6.5] (n = 8)
Blood pressure support during hospitalization, n (%)											
Fluid resuscitation	251 (12.3)	21 (11.0)	38 (10.4)	0	40 (18.6)	6 (2.0)	39 (19.2)	19 (5.7)	33 (11.8)	22 (11.0)	33 (28.9)
Vasopressors	101 (5.0)	21 (11.0)	22 (6.0)	2 (4.0)	3 (1.4)	5 (1.7)	4 (2.0)	11 (9.1)	18 (6.5)	12 (6.0)	3 (2.6)
Invasive procedures	30 (1.5)	6 (3.1)	6 (1.6)	1 (2.0)	1 (0.5)	2 (0.7)	1 (0.5)	1 (0.8)	8 (2.9)	4 (2.0)	0
Isolation required, n (%)	154 (7.6)	10 (5.2)	49 (13.4)	2 (4.0)	14 (6.5)	16 (5.3)	9 (4.4)	5 (4.1)	20 (7.2)	4 (2.0)	25 (21.9)
Mechanical ventilation required during hospitalization, n (%)	280 (13.7)	32 (16.8)	69 (18.9)	5 (10.0)	5 (2.3)	33 (11.0)	23 (11.3)	14 (11.6)	38 (13.6)	41 (20.5)	20 (17.5)
Invasive	139 (6.8)	28 (14.7)	42 (11.5)	2 (4.0)	3 (1.4)	5 (1.7)	16 (7.9)	10 (8.3)	11 (3.9)	16 (8.0)	6 (5.3)
Duration, days, mean (SD) [median]	10.5 (12.7) [6.0] (n = 133)	9.5 (9.2) [7.5] (n = 26)	8.5 (9.2) [6.0] (n = 42)	47.5 (21.9) [47.5] (n = 2)	25.3 (6.4) [28.0] (n = 3)	11.4 (14.6) [5.0] (n = 5)	12.0 (23.2) [3.0] (n = 14)	9.0 (7.7) [7.0] (n = 9)	12.2 (11.1) [8.0] (n = 11)	8.0 (9.4) [5.0] (n = 15)	9.8 (9.9) [8.0] (n = 6)
Non-invasive	166 (8.1)	8 (4.2)	36 (9.8)	5 (10.0)	2 (0.9)	29 (9.7)	8 (3.9)	6 (5.0)	28 (10.0)	30 (15.0)	14 (12.3)
Duration, days, mean (SD) [median]	5.2 (4.5) [4.0] (n = 155)	2.9 (2.0) [3.0] (n = 7)	5.3 (4.3) [4.0] (n = 35)	5.4 (4.0) [4.0] (n = 5)	8.0 (5.7) [8.0] (n = 2)	6.0 (4.9) [5.0] (n = 22)	3.4 (3.5) [2.0] (n = 7)	7.2 (7.1) [5.5] (n = 6)	4.3 (3.3) [3.0] (n = 27)	6.6 (5.9) [4.0] (n = 30)	2.7 (1.4) [2.5] (n = 14)
Parenteral nutrition, n (%)	94 (4.6)	7 (3.7)	38 (10.4)	2 (4.0)	3 (1.4)	10 (3.3)	5 (2.5)	2 (1.7)	6 (2.2)	14 (7.0)	7 (6.1)
Duration of parenteral nutrition, days, mean (SD) [median]	9.1 (10.6) [5.0] (n = 88)	7.2 (9.2) [3.0] (n = 6)	6.4 (4.7) [5.0] (n = 38)	51.5 (7.8) [51.5] (n = 2)	12.5 (10.6) [12.5] (n = 2)	7.7 (6.9) [6.0] (n = 9)	17.8 (15.9) [12.0] (n = 5)	14.5 (9.2) [14.5] (n = 2)	13.7 (13.5) [6.0] (n = 6)	8.7 (10.2) [5.0] (n = 12)	1.8 (0.75) [2.0] (n = 6)
Acute renal failure necessitating renal replacement therapy, n (%)	46 (2.3)	10 (5.2)	6 (1.6)	0	1 (0.5)	1 (0.3)	5 (2.5)	5 (4.1)	7 (2.5)	6 (3.0)	5 (4.4)
Duration of renal failure, days, mean (SD) [median]	6.5 (8.6) [3.0] (n = 37)	4.0 (4.5) [2.0] (n = 8)	10.2 (15.1) [4.0] (n = 5)	–	2.0 (-) [2.0] (n = 1)	11.0 (-) [11.0] (n = 1)	4.5 (2.1) [4.5] (n = 2)	2.5 (1.7) [2.0] (n = 4)	11.0 (13.3) [5.0] (n = 7)	7.2 (4.1) [8.0] (n = 6)	1.7 (0.6) [2.0] (n = 3)
Septic shock, n (%)	84 (4.1)	15 (7.9)	22 (6.0)	–	4 (1.9)	1 (0.3)	4 (2.0)	9 (7.4)	18 (6.5)	6 (3.0)	5 (4.4)
Home-based care, n (%)	73 (3.6)	9 (4.7)	21 (5.7)	1 (2.0)	1 (0.5)	3 (1.0)	14 (6.9)	3 (2.5)	17 (6.1)	1 (0.5)	3 (2.6)

CAP: community-acquired pneumonia; HCAP: healthcare-associated pneumonia; ICU: intensive care unit; SD: standard deviation.

Isolation of the patient was comparatively frequent in the UK and France, while these countries, along with Turkey, also had the highest proportions of patients undergoing mechanical ventilation. On all other measures of resource use, either there were no meaningful differences or the patient numbers involved were too small to make any meaningful comparisons.

### Association of antibiotic treatment modification with increased use of hospital resources

Patients with initial antibiotic treatment modification had a longer mean hospital stay than those without (16.1; median: 12.0 versus 11.1; median: 9.0 days) (Table 3). The unit costs per bed/day in either a secondary-level hospital or a tertiary-level/teaching hospital for each of the participating countries are shown in Table 5. These data were obtained from the WHO-CHOICE database [8] and show the different costs of hospitalization in local currency for each country (US$, Euro, Turkish Lira [TL] or GBP).

**Table 5:** Duration of hospitalization and estimated associated costs in participating countries

**Country**	**Total duration of hospitalization in REACH study, days, median**	Secondary-level hospital*		**Estimated cost of median length of stay in REACH study, US$ (based on secondary-level hospital costs)**	**Tertiary-level/teaching hospital**^**†**^		**Estimated cost of median length of stay in REACH study, US$ (based on tertiary-level hospital costs)**
		**Cost per bed/day, local currency**^**‡**^	**Cost per bed/day, US$**		**Cost per bed/day, local currency**	**Cost per bed/day, US$**	
Belgium	10.0	424.70	624.60	6246.00	549.20	807.64	8076.40
France	11.0	396.00	582.40	6406.40	512.10	753.06	8283.66
Germany	9.0	401.10	589.84	5308.56	518.60	762.69	6864.21
Greece	7.0	266.20	391.42	2739.94	344.20	506.13	3542.91
Italy	11.0	337.80	496.80	5464.80	436.80	642.38	7066.18
The Netherlands	9.0	493.40	725.58	6530.22	638.00	938.21	8443.89
Portugal	10.0	199.50	293.35	2933.50	257.90	379.31	3793.10
Spain	9.5	307.60	452.30	4296.85	397.70	584.85	5556.08
Turkey	10.0	147.00	112.88	1128.80	190.00	145.96	1459.60
United Kingdom	7.0	311.40	576.60	4036.20	402.60	745.56	5218.92

*Secondary-level hospitals = hospitals intended primarily for treating referral cases, with bed size ranging from 200 to 800 beds.

^**†**^Tertiary-level/teaching hospitals = hospitals intended for referral cases, with a teaching component and highly specialised staff and technical equipment, including ICU and bed size ranging from 300 to 1,500 beds.

^‡^Local currency is Euro for all countries except Turkey (Turkish Lira) and United Kingdom (GBP).

Costs are estimates of unit costs for 2007 and 2008 base-year values. They represent costs for public facilities in urban areas that are operating at 80% capacity.

Cost estimates represent only the ‘hotel’ component of hospital costs, excluding the costs of drugs and diagnostic tests but including costs such as personnel, capital and food costs.

## Discussion and conclusions

The REACH study has provided an opportunity to assess real-world clinical management patterns of patients hospitalized with CAP across Europe. Here we present data on the level of resource use associated with this disease in Europe as a whole and in each participating country and consider the implications in terms of the economic burden.

**Figure 1:**
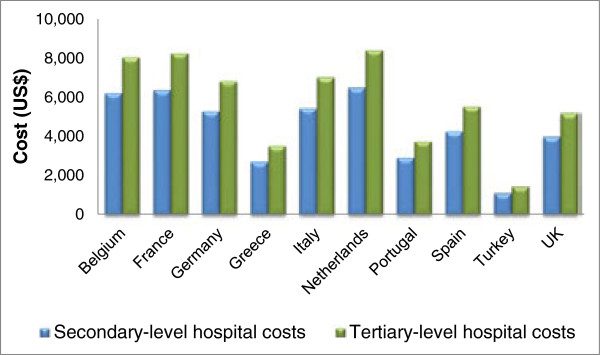
**Estimated cost of median length of stay for patients with CAP in European hospitals.** Secondary-level hospitals = hospitals intended primarily for treating referral cases, with bed size ranging from 200 to 800 beds. Tertiary-level/teaching hospitals = hospitals intended for referral cases, with a teaching component and highly specialised staff and technical equipment, including ICU and bed size ranging from 300 to 1,500 beds.

This study has confirmed that CAP is associated with a high level of resource use. Previous studies show that the key elements of the costs of CAP are hospital stay and antibiotic use [2,4,5]. These findings are supported by our study, where there was a considerable mean length of stay in hospital of 12.6 days (median: 10.6). To assess the impact of this length of stay in monetary terms, we obtained data on the median costs of hospitalization in each of the countries included (2007–2008 data), using the WHO CHOICE project [8] (Table 5 and Figure 1). This project, which states costs in US$, gives information on three different levels of hospital care: primary, secondary and teaching (tertiary) hospitals. Based on the definition of primary hospital (hospitals intended primarily for treatment of simple cases), we excluded this level of care from the analysis and looked only at secondary and teaching hospitals (definitions for each of which can be found in the footnotes beneath Table 5 and Figure 1).

Based on the WHO CHOICE information, the estimated costs for a hospital stay of 10.6 days ranged between US$1,197 (Turkey) and US$7,691 (the Netherlands) in a secondary-level hospital and between US$1,547 (Turkey) and US$9,945 (the Netherlands) in a teaching hospital. Using the specific median lengths of stay that were found in the REACH study for each of the individual countries (Table 5), the estimated costs of a hospital stay ranged between US$1,129 in Turkey and US$6,530 in the Netherlands in a secondary-level hospital and between US$1,460 in Turkey and US$8,444 in the Netherlands in a teaching hospital (Table 5 and Figure 1). These costs are somewhat higher than those reported previously. For example, a prospective observational study over 13 months in 271 patients with CAP hospitalized in a tertiary hospital in Spain found that the median total cost per patient was €1,683 [9]. A prospective cohort study evaluating the costs of CAP in Germany found that the median cost per treated episode of CAP in 580 patients in a prospective open study was US$1,333, of which US$604 were for ‘hotel’ costs and US$426 were for staff costs [2]. However, these studies are limited by their small size and restriction to single countries. The REACH study, conversely, featured patient data from numerous hospitals in ten different countries, suggesting that the data produced are more representative of Europe as a whole. An additional strength of the present analysis was the use of WHO CHOICE cost estimates, which were produced using a robust method applied consistently across all countries.

As expected, initial antibiotic treatment modification was associated with considerable increases in every measure of resource use, including hospital stay, ICU admission, blood pressure support, mechanical ventilation and renal replacement therapy, compared with patients not requiring initial antibiotic treatment modification. However, the causality of the relationships observed cannot be determined from the available data, and it is important to note that certain variables such as parenteral nutrition and renal failure may be influenced by underlying comorbidities. Modification of initial antibiotic treatment was associated with an additional median length of stay in hospital of 3.0 days compared with patients not requiring initial antibiotic treatment modification. Based on the WHO CHOICE costs outlined above, this would represent a considerable increase in costs for hospital stay of between US$339 (Turkey) and US$2177 (the Netherlands) for a secondary hospital and US$438 (Turkey) and US$2815 (the Netherlands) for a tertiary hospital. Of course, increased use of other supporting resources such as mechanical ventilation and renal replacement therapy will have resulted in further increases in costs in patients requiring initial antibiotic treatment modification.

Further support is provided by the analysis of resource use by country, which demonstrated that higher levels of resource use were observed in countries with higher initial antibiotic treatment modification rates. For example, the longest duration of hospital stay (mean 15.0 days) and the highest rate of ICU admissions were observed in Belgium, which had a high initial antibiotic treatment modification rate. A possible alternative explanation for differences in resource use between countries may be differences in healthcare policies. Length of stay, for example, would vary depending on the availability of continuing care outside the hospital environment, and indeed the percentage of patients requiring home-based care showed considerable variation across the different countries. Additional supportive evidence comes from other studies, in which availability of ICU beds and rates of admission to the ICU have been shown to vary widely across different countries [10].

Previous studies show that the costs of ICU treatment are higher than those of acute-ward treatment, with an estimated mean total cost per patient per day of €791 in Germany [11]. These additional costs arise from a number of factors, including use of specific resources such as mechanical ventilation, which had a mean incremental cost in a US study of US$1,522 per day [12]. In our study, higher rates of admission to the ICU were observed in patients with initial antibiotic treatment modification (18.0%) than in those without (11.9%) and in the small subpopulation of patients with recurrent infection (20.2%) than in those without (11.4%).

Considerably higher rates of ICU admission were observed in patients with septic shock (82.1%) than in those without (10.7%). Previous research has shown that complications such as septic shock result in increased costs because of an increased need for diagnostic procedures and monitoring, and for further therapeutic interventions [11]. Indeed, higher rates of mechanical ventilation, as well as blood pressure support, parenteral nutrition and renal replacement therapy, in patients with septic shock versus those without were observed in the present study, confirming these prior results.

Underlying disease characteristics also have a role in determining the level of resource use and associated costs. For example, requirements for resource use in patients with HCAP were different to those in patients with CAP. These results align with those of a previous, comprehensive epidemiological study in the US, which found that mean hospital costs were higher for patients with HCAP than patients with CAP [13].

A key limitation of this analysis is that no cost information was obtained directly from the hospitals enrolled in the study, meaning that any conclusions from a health economic perspective will need careful verification across different health settings. Data on costs of antibiotic treatment would have been interesting. Costs from hospitals in each country may form the basis of further local analyses, which will clarify the relevance of the findings to separate countries and aid understanding of inter-country differences. Low patient numbers were enrolled in certain countries, such as Germany (n = 50), the UK (n = 114) and Portugal (n = 121), reducing the statistical value of analyses in those countries.

The REACH study has highlighted a considerable rate of initial antibiotic treatment modification in patients hospitalized with CAP in Europe [7]. Here we have shown that hospital resource use is high in patients with CAP and that initial antibiotic treatment modification is associated with higher levels of resource use, and associated costs, than are seen in patients without initial antibiotic treatment modification. While the causality of the association between initial antibiotic treatment modification and resource use cannot be determined from the available data, these results suggest that consideration of the influence of initial treatment choices on resource use may be warranted.

## Competing interests

The REACH study was sponsored and funded by AstraZeneca.

HO has received research grants, speaking invitations and conference invitations from Astellas, AstraZeneca, Gilead, MSD, Pfizer and TEVA and consultancy fees from AstraZeneca, Gilead, MSD and TEVA.

JG has received research grants, speaking invitations and conference invitations from Bayer, GSK, AstraZeneca, Novartis, Vifor Pharma, Pfizer and Astellas, and has recent or ongoing consultancies with GSK, Bayer, Pfizer, Novartis, Vifor Pharma, Janssen Cilag, AstraZeneca, Astellas, Theravance and Durata.

FB has received research grants from GSK, Chiesi, Zambon and Pfizer, congress lecture fees from GSK, Chiesi, Pfizer and Abbott and consultancy fees from AstraZeneca, GSK and Pfizer.

JM and EP are employees of AstraZeneca.

KMB has received consultancy fees from Celgene Corporation, AstraZeneca, Worldwide Clinical Trials, Integrium LLC, Cypress Pharmaceuticals, Sigma-Tau Pharmaceuticals, Outcomes Research (now owned by Quintiles), Multiple Myeloma Research Foundation, MedImmune, ACT Oncology and BioSoteria.

## Authors’ contributions

The chief investigators (HO, FB, JG) designed the trial, with input from the sponsor. The chief investigators, together with KM initiated the analysis presented here, with the other investigators, JM and EP contributing to the analysis and interpretation. The decision to submit the report for publication was made by the lead contributors and chief investigators, who drafted and finalised the report with the help of a medical writer. The sponsor funded editorial assistance and reviewed the draft before submission. All authors read and approved the final manuscript.

## Pre-publication history

The pre-publication history for this paper can be accessed here:

http://www.biomedcentral.com/1471-2466/14/36/prepub

## Supplementary Material

Additional file 1 List of participating sites, by country. Click here for file
